# Assessment of Risk for Exposure to Bats in Sleeping Quarters Before and During Remediation — Kentucky, 2012

**Published:** 2013-05-17

**Authors:** Douglas Thoroughman, John Poe, Thursa Sloan, TJ Sugg, Kraig Humbaugh, Jesse Blanton, Elizabeth S. Russell, Ryan M. Wallace

**Affiliations:** Kentucky Dept for Public Health; Div of High-Consequence Pathogens and Pathology, National Center for Emerging and Zoonotic Infectious Diseases; EIS officers, CDC

Bats are a reservoir for rabies viruses and have been identified as the most common source of human rabies infections acquired in the United States. The last human rabies fatality from a bat exposure reported in a Kentucky resident occurred in 1996 (1). In July 2012, the Kentucky Department for Public Health (KDPH) was advised of multiple potential bat exposures following efforts to eliminate a bat colony from a volunteer facility. Bats had routinely been sighted in a brick building in eastern Kentucky that had been used as sleeping quarters by an organization that, since 1999, had hosted thousands of children and adults who performed stints of volunteer work over the course of several days. To assess the risk for bat exposure, KDPH and CDC interviewed 257 (94%) of the 273 volunteers and staff members who had slept in the facility in 2012. As a result of that assessment, 48 (19%) persons were identified as potentially exposed, and 16 (33%) of the 48 were recommended to receive rabies postexposure prophylaxis (PEP), including three persons categorized as at high risk and 13 as at moderate risk for exposure. This report highlights the need for guidelines for appropriate remediation of bat infestation and public health investigations of potential mass bat contacts.

## Assessment of Risk for Bat Exposure

On July 28, 2012, KDPH was notified about potential mass bat contacts at a volunteer facility in eastern Kentucky that occurred before and during remediation efforts to rid the facility of bats. The facility had housed 273 volunteers and staff members during 2012 and was reported to have had a roosting colony of 200–300 big brown bats (*Eptesicus fuscus*). Bats had been seen in and around the facility since 1999; however, an increase in human contact with the bats during 2012 prompted concern. An investigative team including KDPH and CDC staff members developed a telephone survey to assess the risk for bat exposure among those who had slept in the facility. Data collected during the risk assessment included dates slept in the building, rooms slept in, level of bunk slept on, and whether the volunteer or staff member saw bats inside or outside of the building. For observed bats, respondents were asked about the apparent health status of the bats (healthy, injured or ill, or dead), direct contact with bats (bitten, scratched, or touched the bat near the head or mouth), whether they had awakened in a room with a bat, and whether they recalled seeing a bat make contact with other persons who were sleeping. In addition, persons were asked whether they considered themselves to be heavy sleepers, slept with skin exposed, or used any medications, drugs, or alcohol that might cause impaired sensation during sleep (2).

A total of 257 (94%) of 273 volunteers and staff members who had slept in the building in 2012 completed a risk assessment and were categorized as at low, moderate, or high risk for bat exposure. Persons who had no indication of potential bat contact were categorized at low risk, and no additional follow-up was recommended for them. Persons were considered at moderate risk if they slept in a room on the night a bat was sighted and had a self-reported condition that could decrease their awareness of bat contact while sleeping. Persons at high risk were those thought to have had direct skin contact with a bat and who could not definitively rule out a bite or scratch. Persons found to be at moderate or high risk for rabies exposure were referred to their medical provider to discuss PEP.

The 257 persons ranged in age from 13 to 87 years, with a median age of 21 years. Bats were sighted in sleeping quarters on 13 nights during June 19–July 24, 2012. Based on these sightings, 48 (19%) persons were considered potentially exposed to bats while they slept (Figure). Sixteen (6.3%) of the 48 persons were determined to be at elevated risk for rabies exposure: three at high risk and 13 at moderate risk. All 16 were advised to receive PEP. Two of the three persons at high risk for exposure had held a bat without gloves. The third person at high risk was awakened when he rolled onto a bat in his bed and caught bats on two separate occasions without the use of gloves. Another person petted a bat (away from the head or mouth) and was considered at moderate risk. No persons reported bat bites or scratches. Males (four of 118) were more likely than females (none of 139) to have touched a bat. A follow-up survey found that all persons at high risk received PEP, and three of 13 at moderate risk received PEP. Because one volunteer at low risk received PEP, KDPH decided to additionally survey 32 randomly selected persons at low risk who had not been recommended for PEP. Of the 29 who responded to the survey, five consulted a physician, and none received PEP.

In rare instances, clinical rabies has developed ≥1 year after exposure (3). Therefore, persons who slept at the facility in 2011 were mailed a notification letter from the volunteer organization with information regarding bats in the facility, basic information on bats and rabies, and directions to seek medical evaluation for risk assessment if they had direct contact with a bat or other exposure concerns. Persons who had stayed at the facility before 2011 were not contacted.

## Remediation of Bat Infestation

The volunteer organization hired pest control experts on July 9, 2012, 19 days before KDPH was notified of the infestation and human contact. Pest control professionals reported finding a colony of 200–300 big brown bats roosting above the ceiling tiles of the volunteer and staff member sleeping quarters. Initial remediation began on July 10 and consisted of installing netting and wire mesh over building entry points above the female sleeping quarters. On four of the subsequent five nights, bats were seen in the female sleeping quarters. On July 16, external entry points above the male sleeping quarters were blocked with wire mesh and netting, but bats were sighted in male sleeping quarters on six of the subsequent seven nights. Outside netting likely allowed the majority of bats to exit the building, whereas others ventured into the sleeping quarters looking for additional exits. The pest control team removed and replaced 60% of the ceiling tiles because of guano and debris, a possible indicator of the longevity and size of the building infestation.

What is already known on this topic?

Bats are a known reservoir for rabies in the United States. Each year an average of two or three persons die from rabies, and in recent years all domestically acquired human rabies cases have resulted from contact with a rabid bat. Currently no recommendations specifically address mass human exposure to bats, a scenario where levels of potential bat exposure might be difficult to assess.

What is added by this report?

This report found that 19% of persons assessed for bat exposure had slept in a room where a bat was sighted at night, and 33% of those persons reported direct contact with a bat. Seventy-four percent of indoor bat sightings occurred in the 2 weeks the facility remained open following the start of bat exclusion efforts. All three participants assessed at highest risk for bat exposure received postexposure prophylaxis (PEP) in response to this investigation, and three of 13 persons at moderate risk adhered to a recommendation to receive PEP. No persons staying at the facility developed rabies.

What are the implications for public health practice?

Each year, millions of persons sleep in seasonal housing quarters and year-round homes in areas with large bat populations. This report describes what appears to have been an effective method for conducting risk assessments on a large transient population exposed to bats in sleeping quarters. Knowledge of the risks for human-bat contact and appropriate bat exclusion efforts could reduce the potential for human-bat contact.

### Editorial Note

Rabies is an acute, progressive, and fatal encephalitis transmitted to humans by a bite from a rabid animal (4) or infectious saliva or neural tissue that comes in direct contact with open wounds or mucous membranes. Since 2002, the source of infection for 21 of 24 domestic human rabies cases was determined to be a bat (1). In 2011, 7% of bats tested in Kentucky were positive for rabies virus (KDPH, unpublished data, 2012). Rabies PEP is recommended for anyone who has been bitten or scratched by a bat (if the bat is unavailable for testing). In addition, thorough risk assessment should be conducted and PEP considered in situations where a bat is identified in direct proximity to a person who cannot be reasonably sure a bat bite or scratch did not occur, such as someone awaking in a room with a bat or having a condition that might decrease awareness of a bat contact (2). Bat bites and scratches typically are not severe, and history of a known bite was not elicited in approximately half of the reported cases of human rabies attributable to bats (3,5). Bites or scratches from animals should be washed with soap and water immediately, and consultation should be sought with a health-care provider or local health department for any potential exposure to bats.

In this report, repeated bat sightings in sleeping quarters by both staff members and volunteers points to a significant lack of public awareness of the risks of bat exposure and a clear need for education of the general public and organizations that provide sleeping quarters in areas with large bat populations. Educational materials were distributed, informing staff members that bats are a reservoir for rabies and any future bat sightings inside facilities should be reported to the local health department to allow for safe capture and testing of bats and remediation of the facility, as necessary.

Mass bat exposure events should be reported immediately to public health officials, who can advise on proper exclusion techniques, which might vary based on the characteristics of the facility, bat colony size, and season. A common exclusion method is to place netting over building entry points to allow exit of bats, but prevent reentry. Juvenile bats are often unable to exit through this netting, and therefore often explore alternative exits, so exclusion timing should take into account the age of the bat population. CDC recommends that steps be taken when excluding bats from group lodging facilities to ensure that the risk for human contact is not increased for those residing or working in the building as a result of bats seeking alternate exit routes during the process. Although no formal guidelines or validated assessments are available that specifically discuss mass exposure to bats, the assessments of risk categorization conducted during this investigation, along with similar recent investigations in the United States (6), could significantly improve the efficiency and outcomes of future investigations.

## Figures and Tables

**FIGURE:**
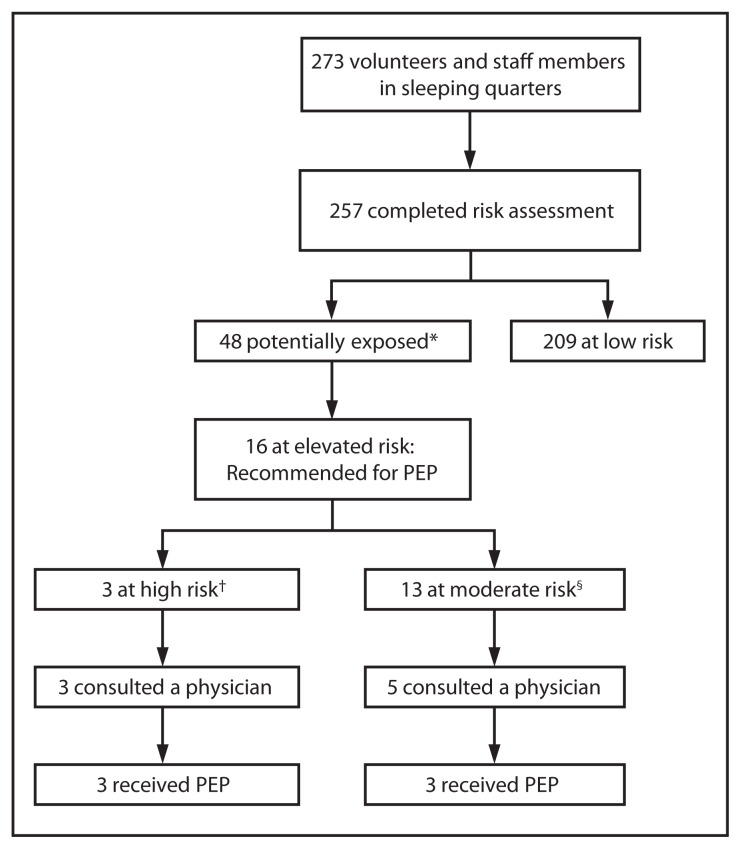
Assessment of risk for bat exposure in a volunteer facility — Kentucky, 2012 **Abbreviation:** PEP = postexposure prophylaxis. * Had direct contact with a bat or slept in a room where a bat was sighted. ^†^ Had direct contact with the mouth or head of a bat or was unable to rule out such contact. ^§^ Had direct contact with a bat other than the mouth or head or was unable to rule out contact with bat while sleeping.
